# Parapharyngeal Neck Schwannomas with Unusual Vascular Displacement

**DOI:** 10.1155/2013/563019

**Published:** 2013-08-26

**Authors:** Kiran Sargar

**Affiliations:** Mallinckrodt Institute of Radiology, Washington University School of Medicine in St. Louis, MO, USA

## Abstract

This case report illustrates two unusual cases of parapharyngeal schwannomas mimicking carotid body tumors in terms of characteristic vascular displacement. Carotid body tumors classically cause splaying of internal and external carotid arteries demonstrating the Lyre sign on imaging. Also interestingly, both of these cases were seen in younger ages and include cervical sympathetic chain schwannoma and vagal schwannoma. However, these schwannomas revealed hypovascularity on imaging studies allowing differentiation from hypervascular carotid body tumors. Preoperative distinction between carotid body tumors and schwannomas is very important.

## 1. Introduction

Carotid body tumors, cervical sympathetic chain schwannomas, and vagal schwannomas have common location in neck that is the retrostyloid compartment of parapharyngeal space [1–3]. Among these, vagal schwannomas are most common followed by carotid body tumors [1, 3]. Various imaging features had been described to differentiate them, which consist of typical displacement pattern of adjacent vessels and internal vascularity. This case report includes two histopathologically proven rare cases of cervical sympathetic schwannoma and vagal schwannoma having similar vascular displacement pattern as that of carotid body tumor. However, both of these tumors revealed hypovascularity on imaging unlike hypervascular carotid body tumors.

## 2. Case One

**Figure 1:**
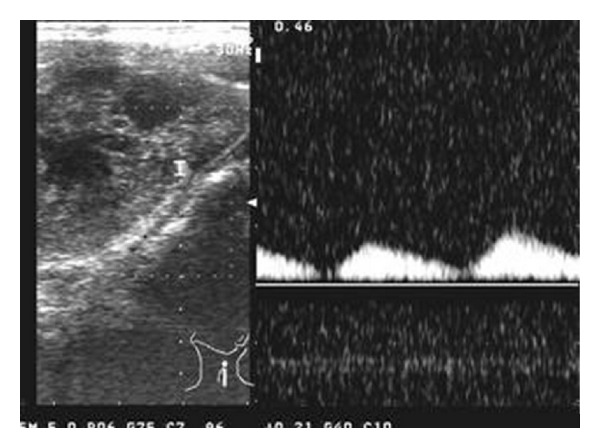
The Doppler ultrasonography of neck reveals a heterogeneous predominantly hypoechoic mass on the left carotid space showing mild peripheral vascularity.

**Figure 2:**
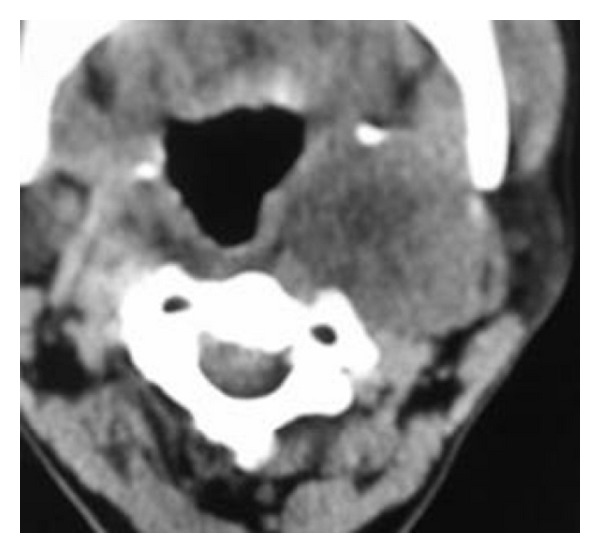
Computed tomography of the neck shows a heterogeneously enhancing hypodense mass on the left carotid space causing splaying of carotid bifurcation.

**Figure 3:**
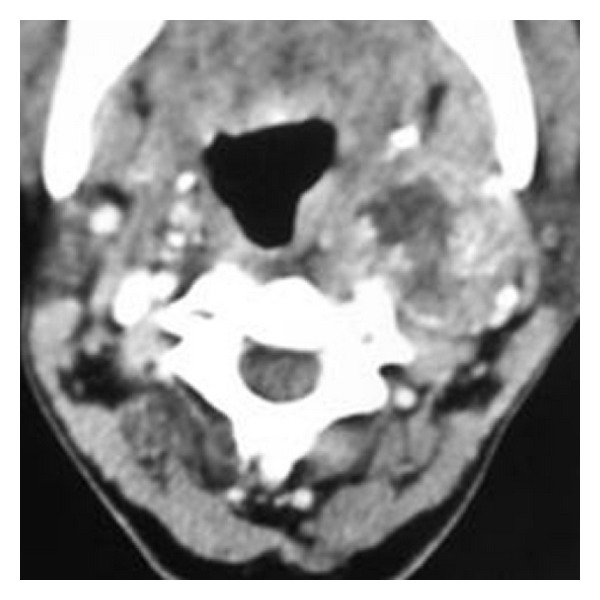
Computed tomography of the neck shows heterogeneously enhancing hypodense mass in left carotid space causing splaying of carotid bifurcation.

**Figure 4:**
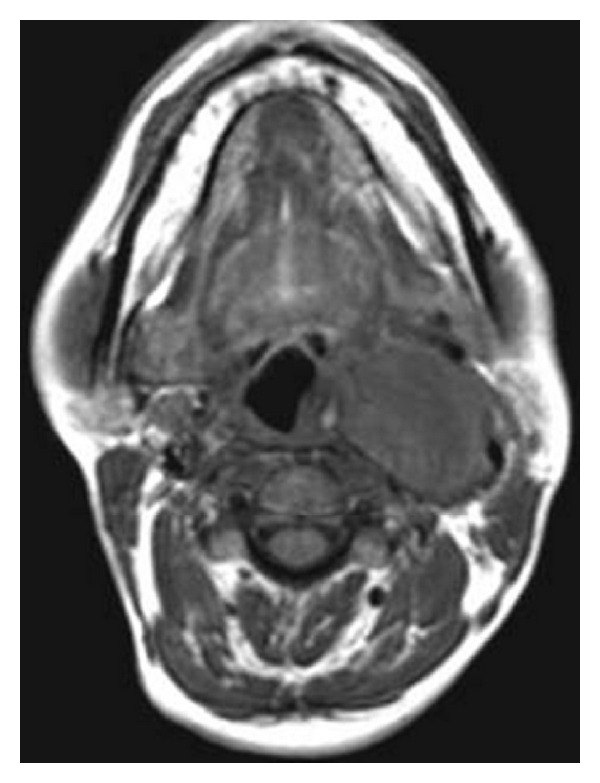
T1-weighted MR image reveals isointense mass on the left carotid space.

**Figure 5:**
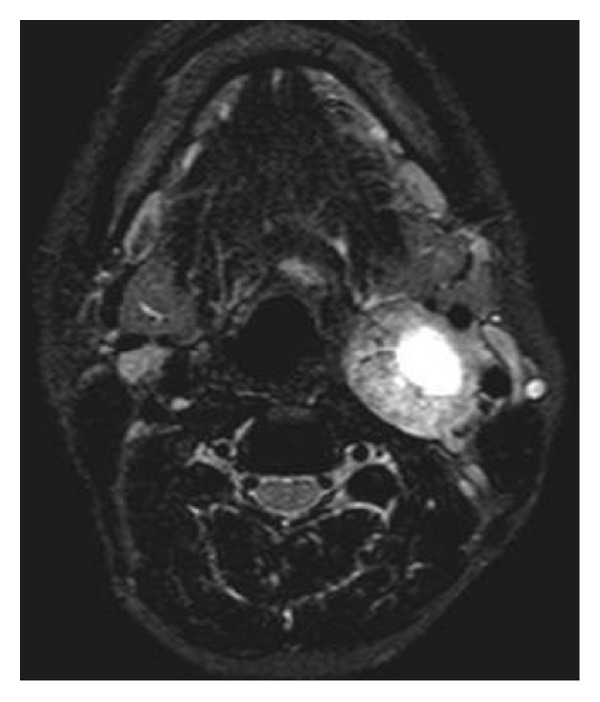
T2-weighted MR image shows hyperintense mass with central cystic component.

**Figure 6:**
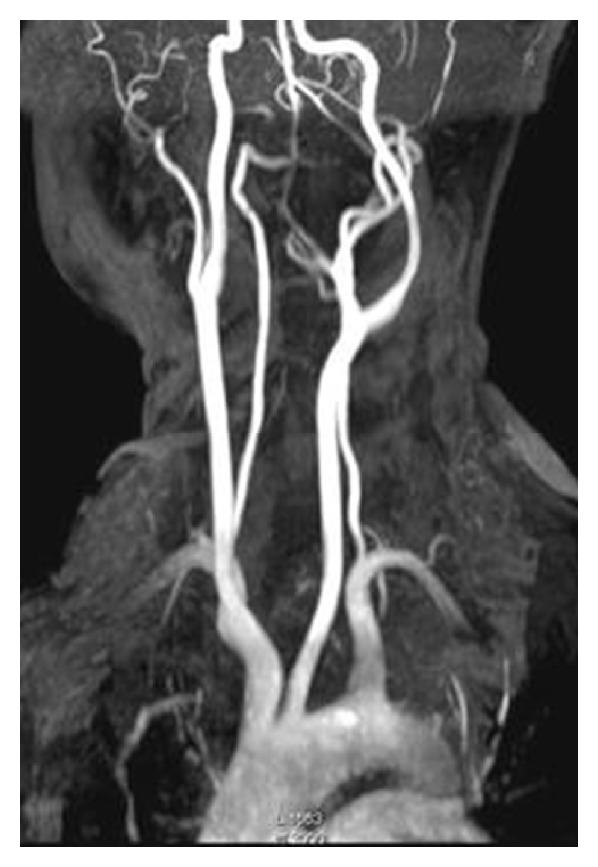
MR angiography image is showing splaying of external and internal carotid vessels on the left side.

**Figure 7:**
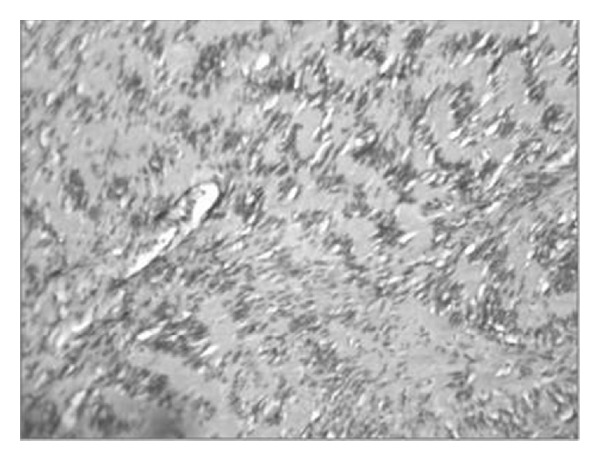
Histopathology slide of the surgical specimen is consistent with diagnosis of Antoni A schwannoma.

An 18-year-old female was referred to the otolaryngology department with a swelling on the left side of the neck since 4 months. On examination, nonpulsatile mass near angle of the left mandible was seen. Cranial nerve examination was normal. Doppler ultrasonography revealed hypoechoic mass in the left carotid space showing mild peripheral vascularity (Figure 1). Computed tomography of the neck showed a heterogeneously enhancing hypodense mass in the left carotid space causing splaying of carotid bifurcation (Figures 2 and 3). On MRI examination, the mass was predominantly hypointense on T1- and hyperintense on T2-weighted images (Figures 4 and 5). Also MR angiography confirmed splaying of external and internal carotid vessels on the left side (Figure 6). The patient underwent surgery. At surgery encapsulated mass was found at the carotid bifurcation extending posteriorly which was separated by blunt dissection from carotid vessels. Vagal and glossopharyngeal nerves were separately seen from the mass. At surgery, the tumor was easily peeled off from carotid and jugular vessels without significant bleeding. Histopathology confirmed diagnosis of cervical sympathetic chain schwannoma with distinct Antoni A areas (Figure 7). Postoperatively patient developed Horner's syndrome on the left side which recovered 3 months after surgery.

## 3. Case Two 

**Figure 8:**
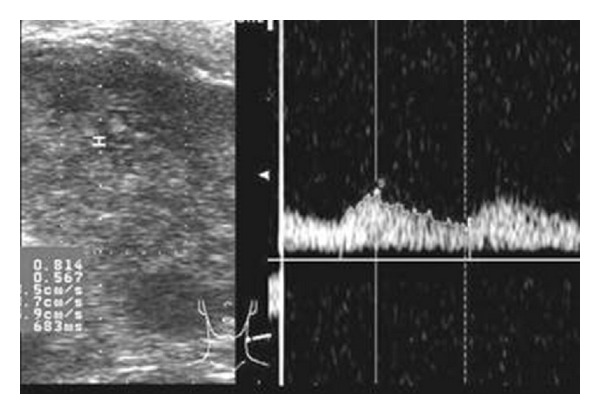
On USG neck, hypoechoic mass is seen on the left parapharyngeal space showing mild vascularity on Doppler study.

**Figure 9:**
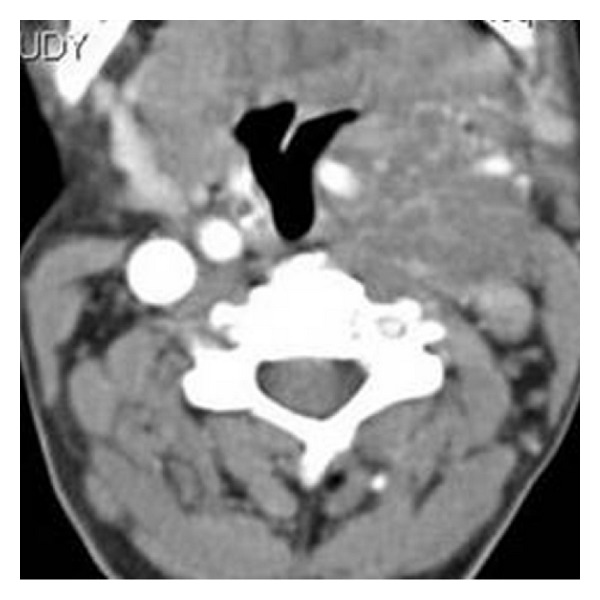
On CT scan, neck with contrast reveals a predominantly isodense mass on the left parapharyngeal space showing mild heterogeneous contrast enhancement. Note the insinuation of the mass between internal and external carotid arteries with resultant splaying of internal and external carotid arteries.

**Figure 10:**
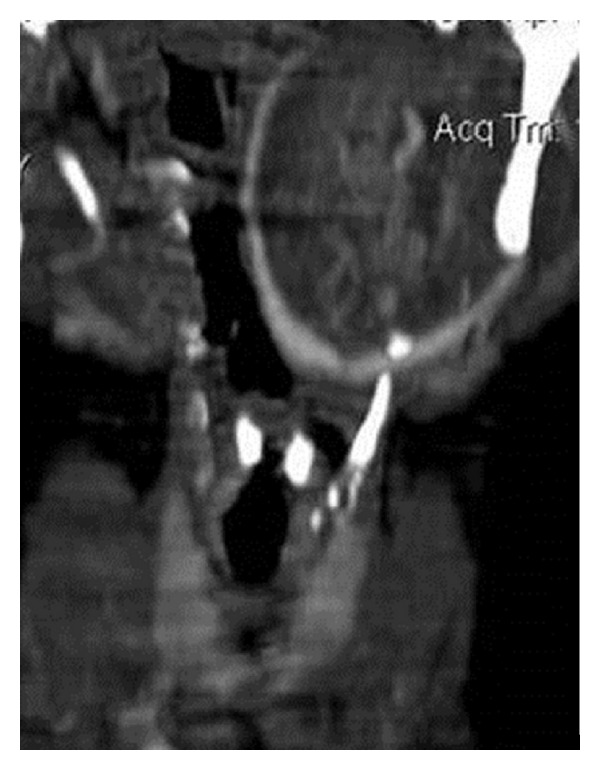
Coronal reconstructed CT image demonstrates splaying of carotid bifurcation by the mass.

**Figure 11:**
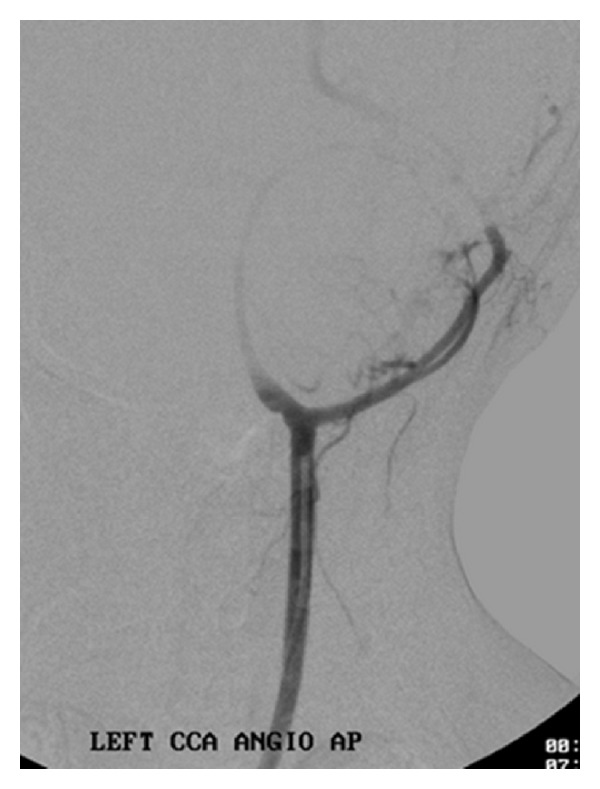
Digital subtraction angiography reveals hypovascular mass causing splaying of internal and external carotid arteries.

**Figure 12:**
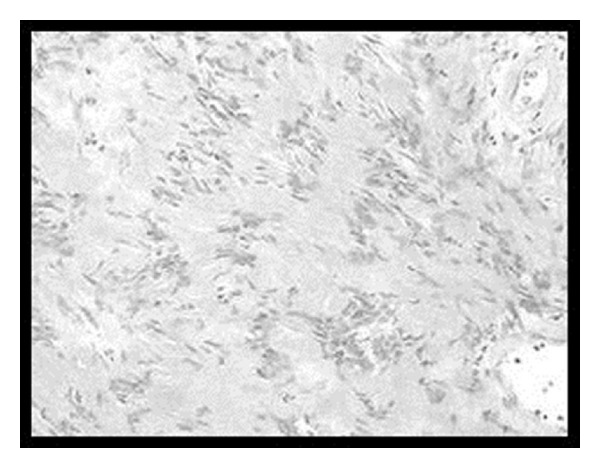
Histopathology slide of the surgical specimen is consistent with diagnosis of Antoni B type of schwannoma.

A 17-year-old female came with history of painless swelling on the left side of the neck and change in voice since 2 months. On examination, there was nontender swelling noted posterior to the angle of the left mandible. USG neck revealed hypoechoic mass on the left parapharyngeal space showing mild vascularity on the Doppler study (Figure 8). On CT scan, large heterogeneous predominantly isodense to hypodense mass was seen in the left parapharyngeal space showing mild heterogeneous contrast enhancement (Figure 9). Mass was seen insinuating between internal and external carotid arteries with resultant splaying of internal and external carotid arteries (Figure 10). Digital subtraction angiography revealed hypovascular mass causing splaying of internal and external carotid arteries (Figure 11). The patient underwent surgery. Intraoperatively, tumour was seen arising from the left vagus nerve and was separating internal and external carotid arteries. On histopathology, mass was proven to be vagal schwannoma (Figure 12).

## 4. Discussion

Vagal schwannomas, carotid body tumours and cervical sympathetic chain schwannomas are the most common tumours of the retrostyloid parapharyngeal space [1, 2]. Carotid body tumors are rare neoplasms, although they represent about 65% of head and neck paragangliomas [1, 2]. These tumors are derived from embryonic neural crest cells and arise in the chemoreceptoric, nonchromaffine paraganglia at carotid bifurcation [1–3]. Epidemiologically they are common in young people, with female preponderance [2, 4]. Since these tumours are located at carotid bifurcation they typically cause splaying of internal and external carotid arteries which can be assessed with CT, MRI, Digital subtraction angiography and ultrasound colour Doppler [1]. This characteristic feature of carotid body tumors helps to distinguish it from other retro styloid pharyngeal mass lesions [1]. Carotid body tumours are hypervascular and enhance intensely on contrast enhanced CT and MRI scans [1].

Schwannomas or neurilemomas, are derived from schwann cells. They are common in middle age with female preponderance [1–3]. In the parapharyngeal space, common sites of the origin of schwannomas are the vagus nerve and the sympathetic chain. Anatomically, vagus nerve is situated within the carotid sheath posterior to the internal carotid artery. As a result, vagal schwannomas typically displace internal carotid artery anteriorly and medially [1, 3, 5, 6]. Cervical sympathetic chain is situated along medial and posterior borders of the carotid sheath, so cervical sympathetic chain schwannomas usually displace internal carotid artery anteriorly [1, 3]. On CT and MRI, both vagal and cervical sympathetic chain schwannomas are hypodense or isodense to the muscles and may have internal cystic areas. Usually, these tumors are hypovascular and show mild contrast enhancement [1–3]. However, sometimes, these tumors also tend to be hypervascular and can show moderate heterogeneous enhancement [1]. On MRI, these masses are hypointense on T1-weighted images and hyperintense on T2-weighted images [1]. 

Preoperative diagnosis is very important in these retrostyloid parapharyngeal masses as management of carotid body tumors varies from surgery to radiation to observation, while complete surgical excision is the therapy of choice in vagal and cervical sympathetic schwannomas [2]. Also, risk of intraoperative hemorrhage is high in carotid body tumors. In carotid body tumor surgery is preferred in younger patients, and radiation is reserved for the elderly or unresectable cases [2].

Both of the described tumors displayed splaying of external and internal carotid arteries on CT contrast study, MRI, and DSA which was also confirmed at surgery. However both of these tumors demonstrated mild vascularity on the Doppler USG, CT, and DSA.

Although characteristic vascular displacement, that is, splaying of carotid bifurcation helps to distinguish carotid body tumor from other retrostyloid parapharyngeal masses, similar vascular displacement can be seen in vagal and cervical sympathetic chain schwannomas. So, this imaging pitfall should be taken into account while considering differential diagnosis of retrostyloid parapharyngeal tumors.

Both of these tumors showed hypovascularity, hence favor schwannomas rather than carotid body tumors. Internal vascularity and enhancement characteristics of the tumor should be given more importance while differentiating schwannomas from carotid body tumors. Hence enhancement pattern is the imaging pearl in differentiating carotid body tumors from schwannomas.
